# 

**Published:** 1906-12

**Authors:** 


### ATLANTA JOURNAL-RECORD OF MEDICINE.

**Successor to Atlanta Medical and Surgical Journal, Established 1855, and Southern Medical Record, Established 1870.**

VOL. VIII. ATLANTA, GA., DECEMBER, 1906. No. 9.

